# Carveol Attenuates Seizure Severity and Neuroinflammation in Pentylenetetrazole-Kindled Epileptic Rats by Regulating the *Nrf2* Signaling Pathway

**DOI:** 10.1155/2021/9966663

**Published:** 2021-08-11

**Authors:** Arooj Mohsin Alvi, Lina Tariq Al Kury, Abdullah Alattar, Ikram Ullah, Asmaa Jan Muhammad, Reem Alshaman, Fawad Ali Shah, Arif Ullah Khan, Jinxing Feng, Shupeng Li

**Affiliations:** ^1^Department of Neonatology, Shenzhen Children Hospital, Shenzhe, China; ^2^Riphah Institute of Pharmaceutical Sciences, Riphah International University, Islamabad 42000, Pakistan; ^3^College of Natural and Health Sciences, Zayed University, Abu Dhabi 49153, UAE; ^4^Department of Pharmacology and Toxicology, Faculty of Pharmacy, University of Tabuk, Tabuk 71421, Saudi Arabia; ^5^Center for Interdisciplinary Research in Basic Sciences, International Islamic University Islamabad, Pakistan; ^6^State Key Laboratory of Oncogenomics, School of Chemical Biology and Biotechnology, Shenzhen Graduate School, Peking University, Shenzhen, China

## Abstract

Epilepsy is a neurodegenerative brain disorder characterized by recurrent seizure attacks. Numerous studies have suggested a strong correlation between oxidative stress and neuroinflammation in several neurodegenerative disorders including epilepsy. This study is aimed at investigating the neuroprotective effects of the natural compound carveol against pentylenetetrazole- (PTZ-) induced kindling and seizure model. Two different doses of carveol (10 mg/kg and 20 mg/kg) were administered to male rats to determine the effects and the effective dose of carveol and to further demonstrate the mechanism of action of nuclear factor E2-related factor (*Nrf2*) in PTZ-induced kindling model. Our results demonstrated reduced levels of innate antioxidants such as superoxide dismutase (SOD), catalase, glutathione-S-transferase (GST), and glutathione (GSH), associated with elevated lipid peroxidation (LPO) and inflammatory cytokines level such as tumor necrosis factor-alpha (TNF-*α*), and mediators like cyclooxygenase (COX-2) and nuclear factor kappa B (NF*κ*B). These detrimental effects exacerbated oxidative stress and provoked a marked neuronal alteration in the cortex and hippocampus of PTZ-intoxicated animals that were associated with upregulated *Nrf2* gene expression. Furthermore, carveol treatment positively modulated the antioxidant gene *Nrf2* and its downstream target *HO-1*. To further investigate the role of *Nrf2*, an inhibitor of *Nrf2* called all-trans retinoic acid (ATRA) was used, which further exacerbated PTZ toxicity. Moreover, carveol treatment induced cholinergic system activation by mitigating acetylcholinesterase level which is further linked to attenuated neuroinflammatory cascade. The extent of blood-brain barrier disruption was evaluated based on vascular endothelial growth factor (VEGF) expression. Taken together, our findings suggest that carveol acts as an *Nrf2* activator and therefore induces downstream antioxidants and mitigates inflammatory insults through multiple pathways. This eventually alleviates PTZ-induced neuroinflammation and neurodegeneration.

## 1. Introduction

Epilepsy, which affects nearly 65 million people worldwide, is among the most prevalent neurodegenerative disease after stroke [1]. It is a syndrome characterized by various neurological conditions, including recurrent epileptic seizures, cognitive deficits, behavioral impairments, and electroencephalographic changes. Epilepsy is most often aggravated by psychiatric comorbidities, like memory and learning deficits, which affect about 30% of the population [2]. Although transient abnormal cortical nerve stimulation can lead to seizure formation, there are numerous other contributing factors having a role in seizure generation, such as excitotoxicity, mitochondrial dysfunction, altered cytokine levels, oxidative and endoplasmic reticulum stress, and genetic factors [3–5]. The clinical use of existing antiepileptic drugs (AEDs) is compromised due to frequent onset of adverse effects along with chronic toxicities of the vital organs [6–8]. Therefore, an in-depth understanding of the mechanisms underlying this disease is required to develop alternative better treatment choices.

Multiple studies have proposed that oxidative stress and inflammation can exacerbate the severity of epilepsy [9, 10] as a consistently high level of inflammatory cytokines is found in seizure pathophysiology [11]. These anomalies can lead to numerous functional and biochemical alterations, including lipid peroxidation (LPO), BBB disruption, and angiogenesis [12, 13]. Moreover, the surge in cytokines is directly related to the permeation of leukocytes and granulocytes to the brain from the surrounding vasculature [14]. Furthermore, resident glial cells are rapidly activated and trigger the release of proinflammatory cytokines such as interleukin-1 (IL-1*β*), tumor necrotic factor-alpha (TNF-*α*), and interleukin-6 (IL-6), which clinically compromise the prognosis of epilepsy [15, 16]. Pentylenetetrazole (PTZ), which is a GABA receptor antagonist, is a widely accepted and established model for inducing epileptic seizures via blockade of the gamma-aminobutyric acid (GABA) receptor, a major inhibitory neurotransmitter in the brain [17]. PTZ exerts a convulsant effect similar to that of human absence seizure and can be used for generating rodent epileptic model [18–20]. Multiple studies have suggested elevated NO levels and reduced antioxidant activity in the rat brain after PTZ treatment [21, 22]. Therefore, maintaining low ROS/RNS levels in the brain is crucial for normal cellular function as it facilitates ablation of subsequent neuroinflammation [23].

Nuclear factor erythroid 2-related factor 2 (*Nrf2*, or NFE2L2) is critically involved in the natural cellular defense system, as it governs the gene expression of numerous antioxidant proteins and ROS-eliminating enzymes, thereby preventing ROS-induced neuronal and cellular damage [24]. Upon activation, *Nrf2* activates the innate antioxidant cellular machinery and upregulates multiple inducible antioxidant enzymes, including heme-oxygenase-1 (*HO-1*), NAD(P)H quinone oxidoreductase 1 (NQO1), glutathione peroxidase (GPx), catalase, and superoxide dismutase (SOD) [25]. There is evidence suggesting the crosstalk between *Nrf2* and NF-*κ*B, which reveals the mechanism through which activated *Nrf2* exerts an anti-inflammatory effect [26–29]. Moreover, previous studies demonstrated the neuroprotective role of *Nrf2* not only in laboratory animals but also in human brain samples [30–32]. We have previously shown that activation of *Nrf2* signaling attenuated infarction area and inflammatory-related pathologies not only in stroke but also in the depression model [33–35]. Therefore, *Nrf2* may be a suitable therapeutic target for managing epilepsy and seizures.

**Figure 1:**
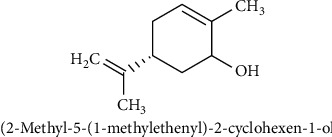
Structure of carveol.

Natural moieties are an attractive source of new drugs, owing to their rich antioxidant potential. Several natural drugs have shown protective potential against a variety of mediators, including free radicals and inflammatory factors [36, 37]. Carveol is a natural monocyclic monoterpenoid antioxidant compound (Figure 1) that is abundant in caraway seeds, mandarin, black tea, dill, and essential oils of orange peel [38, 39]. Carveol has been reported in traditional Chinese medicine as an antispasmodic, carminative, astringent, and further used for indigestion and dyspepsia [40]. We previously demonstrated the neuroprotective potential of carveol in ischemic brain injury by attenuating infarction area [33]. In another study, carveol mitigated hepatocellular necrosis by showing antioxidant, antihyperlipidemic, and anti-inflammatory activities [41]. Furthermore, carveol exhibited promising results in the management of diabetes [42]. Given the strong antioxidant potential of carveol and its promising properties, this study is aimed at evaluating whether carveol administration can ameliorate PTZ-induced epileptogenesis in a rat model by modulating the *Nrf2* pathway.

## 2. Materials and Methods

### 2.1. Chemicals and Reagents

Carveol (#192384, PubChem ID:24851543), a mixture of isomers, with 97% purity, and 3,3-diaminobenzidine tetrahydrochloride hydrate (#D5637, PubChem ID:57654109) were purchased from Sigma-Aldrich (USA). Mouse monoclonal anti-p-NF-*κ*B (SC-271908), mouse monoclonal anti-TNF-*α* (SC-52B83), mouse monoclonal anti-*HO-1* (SC-136960), rabbit polyclonal anti-Nrf2 (SC-722), mouse monoclonal anti-VEGF (SC-7269), and ABC Elite kit (SC-516216) were purchased from Santa Cruz Biotechnology, Dallas, TX, USA). Rat ELISA kits p-NF-*κ*B (SU-B28069) and TNF-*α* ELISA kit (SU-B3098) were procured from Shanghai Yuchun Biotechnology, Shanghai, China, while rat COX-2 (E-EL-M0959) was purchased from Elabscience Biotechnology Inc., Houston, TX, USA. The horseradish peroxidase-conjugated secondary antibody (ab-6789) was obtained from Abcam (Cambridge, UK). Proteinase K (#02193981-CF) was obtained from MP Bio USA. All other solvents and reagents as DPX Mounting media (#06522), 5,5′-dithiobis (2-nitrobenzoic acid) (DTNB, #D8130, PubChem ID:24894189), trichloroacetic acid (TCA, #T6399, PubChem ID:24900373), and N-(1-naphthyl) ethylenediamine dihydrochloride (#222488, PubChem ID:24853334) were procured from Sigma-Aldrich (St. Louis, MO, USA).

### 2.2. Animals and Ethical Approval

Adult male Sprague-Dawley rats (weight 250–300 g) were habituated under laboratory conditions at 25°C for 7 days, with 12 h alternating light and dark cycles; moreover, they received standard commercial diet and water ad libitum. All experimental procedures were conducted following the ARRIVE guidelines and approved by the Research and Ethical Committee (REC) of the Riphah Institute of Pharmaceutical Sciences (Approval ID: Ref. No. REC/RIPS/2018/14; date of approval: November 15, 2018).

### 2.3. Acute Toxicity Testing

To determine the acute toxicity of the selected natural compound, we included 10 nonpregnant nulliparous female rats and divided them into the control and treatment groups (each *n* = 5). After being deprived of food and water overnight, one rat was administered a limited oral dose of 2000 mg/kg per OECD guidelines 425 on the next day [43, 44]. After being observed for 24 h and survival being confirmed, the same protocol was followed for the remaining rats in the treatment group. They were initially observed for 48 h for any signs of distress and mortality; subsequently, they were observed daily for 14 days for other toxicity signs, including squinted eyes, writhing, salivation, tremors, convulsions, loss of fur, change in overall behavior, stress, and mortality. On the 15^th^ day, blood samples were obtained from animals via cardiac puncture for various biochemical analyses, including wet organ weight, antioxidant profile, liver function tests, renal function tests, and hematological profile. Subsequently, the animals were sacrificed under anesthesia, and vital organs were collected for histopathological examination.

### 2.4. Seizure Induction Using PTZ

Seizures were induced as previously described, with slight modifications [45, 46]. Briefly, PTZ was dissolved in normal saline and intraperitoneally (IP) injected into the PTZ-kindled group at a subconvulsive dose of 40 mg/kg at 48 h intervals for 15 days until they were fully kindled and showed stage 5 or 6 on three consecutive injections. Only successfully kindled animals were included in the study.

### 2.5. Study Design and Animal Treatment

**Figure 2:**
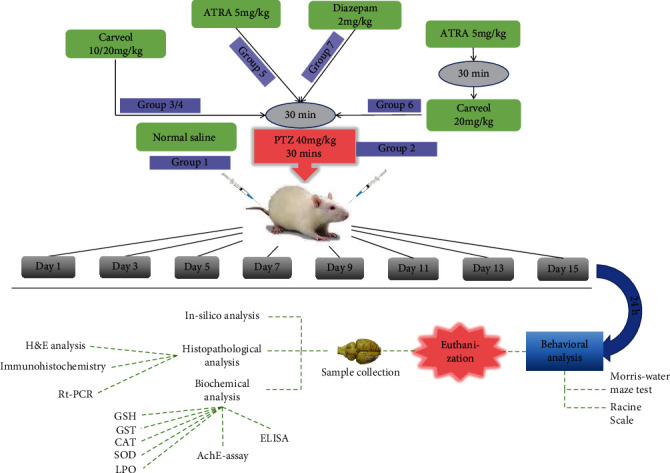
Diagrammatic illustration of the experimental protocol. The treatment protocol was performed for 15 days. In all these groups, a loading dose of PTZ (40 mg/kg, IP) was injected 30 mins after drug treatment (ATRA, carveol, or diazepam), except in the saline group. Brain tissues were collected after 24 h of the last dose for further analysis.

Animals were randomly divided into seven groups (*n* = 10, each group) as follows: group 1 (control group): saline injection containing 5% DMSO were administered at 48 h intervals for 15 days; group 2 (PTZ control group): 40 mg/kg PTZ administered until stage 5 convulsions, with eight injections being administered; group 3/4 (treated group): rats received protective doses of carveol 10 (Car-10) and 20 mg/kg (Car-20) and were administered 30 min before PTZ; group 5 (ATRA+PTZ group): rats were treated with 5 mg/kg all-trans retinoic acid (ATRA) and were administered 30 min before PTZ; group 6 (ATRA+PTZ+Car): rats were treated with ATRA 30 mins before giving carveol and PTZ was administered 30 mins after carveol treatment, followed by behavior recording for 30 min; group 7 (standard group): rats were treated with 2 mg/kg diazepam at 30 min before PTZ administration. Carveol, ATRA, PTZ, and diazepam were dissolved in normal saline containing 5% DMSO and were administered for 15 days at a 48 h interval (Figure 2). Notably, the selected carveol dose was determined in a previous study using a neurodegenerative model established in our lab [33].

### 2.6. Evaluation of Behavioral Characteristics

#### 2.6.1. Racine's Scale

Seizure activity was evaluated for 30 min after each PTZ administration. Behavioral characteristics, including latency, intensity, and convulsion stage, were recorded for 30 min after each PTZ dose using the modified Racine scale [47]: stage 0—no response; stage 1—hyperactivity, restlessness, and vibrissae twitching; stage 2—head nodding, head clonus, and myoclonic jerks; stage 3—unilateral or bilateral limb clonus; stage 4—forelimb clonic seizures; stage 5—generalized clonic seizures with falling; stage 6—hind limb extensor; and stage 7—death (Table 1). We calculated the mean seizure intensity by taking the mean of all individual seizure scores and dividing them by the number of animals, followed by plotting against the treatment duration. Seizure latency was measured as the duration between PTZ administration and the appearance of the first clonic seizure, jerky body movement, or sudden twitch. Seizure frequency was calculated as the number of seizures experienced by the animal within 30 min of PTZ administration, regardless of the seizure stage. Animals were considered kindled when they reached stage 5 (clonic-tonic seizures) or 6, after three consecutive PTZ injections at 48 h intervals. The investigator who performed behavioral trials was blinded from the group allocation to avoid any bias.

#### 2.6.2. Morris Water Maze (MWM) Test

The MWM test was performed to assess the cognitive deficits and spatial learning ability of the rats as previously discussed [47]. The MWM is comprised of a circular pool with a height and diameter of 50 cm and 120 cm, respectively. The pool was hypothetically divided into four quadrants with reference to the target quadrant. The quadrant where the probe or the elevated platform (placed 1 cm beneath the water) was placed was tagged as the target quadrant and then the right-left and opposite quadrant. The water temperature was maintained at 25°C ± 1°C, the position of the platform was fixed, and each time, rats were dropped from different quadrants. A blind observer recorded the escape latency period, i.e., the time taken by the rat to locate and climb the raised platform. The experiment lasted for four days. In the training sessions, the rats were trained to locate and climb the raised platform with a staying time of 5-7 s. The observer recorded the time when the animal was dropped into the water, and if the rat failed to locate the platform within 90 s, the observer manually guided the rat to the platform. The training session was conducted twice a day at 25 min intervals. Similarly, the escape latency interval for each rat was observed and recorded in each training session during the three days of the testing sessions. A decrease in escape latency was considered to indicate neurodegeneration.

On the last day of behavior analysis, we performed a probe test to check the spatial memory. The probe was removed from the target quadrant, and rats were dropped opposite to the target quadrant, with the time spent in each quadrant being recorded for 60 s. The percentage of time spent in the target quadrant was considered a measure of the extent of neurodegenerative potential and memory function.

### 2.7. Tissue Collection and Histological Preparation

At 24 h after the last PTZ administration, the rats were quickly decapitated, and their brains were removed on an ice-cold glass plate. The hippocampus and cortex were separated; subsequently, half of the samples were stored at -80°C for biochemical processing while the other half was kept in 4% formalin solution for histopathological and immunohistochemical analysis. Brain tissue samples were homogenized using 0.1 M sodium phosphate buffer (pH 7.4) containing phenylmethylsulfonyl fluoride (PMSF) as a protease inhibitor. Subsequently, samples were centrifuged at 4000×g for 10 min at 4°C, and the supernatant was used for various biochemical assays.

### 2.8. Estimation of Brain Acetylcholinesterase (AChE) Activity

Brain AChE activity was determined as previously described by Ellman et al. (1961), with slight modifications [48]. Briefly, 100 *μ*L of DTNB reagent (0.1 mM) was added to 2.6 mL of phosphate buffer (pH 7.4); subsequently, 0.4 mL of brain tissue homogenate was added to this mixture. The initial reading of this reaction mixture was taken at 412 nm before substrate addition. Next, 20 *μ*L of the substrate (acetylcholine iodide, 1 mM) was added to this mixture, and the absorbance was recorded every 10 min for 20 min. The mean change in the absorbance was calculated as follows:

(1) $$\begin{array}{c} R=5.74\times 10-4\times \frac{A}{{\text{C}}_{\text{o}}}, \end{array}$$

*A* = change in absorbance per minute.

*R* = rate of moles of acetylthiocholine iodide hydrolyzed per min/g of brain tissue.

*C*_o_ = original concentration.

Enzyme activity was expressed as *μ*moles of acetylcholine hydrolyzed per milligram of protein.

### 2.9. Antioxidant Assays

#### 2.9.1. Reduced Glutathione (GSH) Activity

GSH was determined to estimate the degree of PTZ-induced oxidative damage and the resulting effect of carveol as previously discussed [34]. We mixed 0.2 mL of the tissue supernatant with 2 mL of DTNB mixture, followed by the addition of 0.2 M phosphate buffer to yield a final volume of 3 mL. The absorbance was measured after 10 min using a spectrophotometer at 412 nm, where phosphate buffer and DTNB solution were used as a blank and control, respectively. The real absorbance value was obtained by subtracting the absorbance of the control from that of the tissue lysate. The final GSH activity was expressed in units of *μ*mol/mg of protein.

#### 2.9.2. Glutathione-S-Transferase (GST) Activity

To calculate GST activity, we freshly prepared 1 mM CDNB and 5 mM GSH solutions in 0.1 M phosphate buffer. Three replicates of the 1.2 mL reaction mixture were kept in glass vials, followed by the addition of 60 *μ*L of tissue homogenate to each of these mixtures. The blank contained water rather than tissue lysate. Next, 210 *μ*L aliquots from the reaction mixture were pipetted out in a microtiter plate; further, absorbance was measured at 340 nm for 5 min at 23°C using an ELISA plate reader (BioTek ELx808, Winooski, VT, USA). GST activity was expressed in units of *μ*mol of CDNB conjugate/min/mg of protein [49, 50].

#### 2.9.3. Superoxide Dismutase (SOD) Activity

We mixed 0.1 mL of tissue homogenate with 0.1 mL of pyrogallol solution (1 M) and 2.8 mL of 0.1 M potassium phosphate buffer (pH 7.4), which yielded a reaction mixture of 3 mL. The absorbance was measured at 312 nm [51]. SOD activity was expressed in U/mg of protein.

#### 2.9.4. Catalase (CAT) Activity

We added 0.05 mL of tissue homogenate to 1.95 mL of phosphate buffer (50 mM, pH 7) and 1 mL of H_2_O_2_ solution (30 mM). The absorbance of the final mixture was measured at a wavelength of 240 nm. The catalase activity was calculated using the following formula:

(2) $$\begin{array}{c} \text{CAT}=\delta \text{O}.\text{D}÷E\times \text{Volume of sample}\left(\text{mL}\right)\times \text{protein}\left(\text{mg}\right), \end{array}$$

where *δ*O.D represents the change in absorbance per minute and *E* represents the extinction coefficient of H_2_O_2_ with a value of 0.071 mmol cm^−1^ [51]. The Lowery method was used to measure protein levels. Catalase activity was expressed as *μ*mol of H_2_O_2_/min/mg of protein.

#### 2.9.5. Determination of Lipid Peroxidation (LPO)

The extent of LPO was estimated by detecting thiobarbituric acid reactive substances (TBARS), as previously described with slight modifications [52]. The assay mixture contained 580 *μ*L of phosphate buffer (0.1 M, pH 7.4), 200 *μ*L of supernatant, 20 *μ*L of ferric chloride, and 200 *μ*L of ascorbic acid (100 mM). The mixture was incubated in a water bath at 37°C for 60 min. Next, the reaction was stopped by adding 1000 *μ*L of trichloroacetic acid (10% TCA) and 1000 *μ*L of thiobarbituric acid (0.66% TBA) to the samples. The tubes were kept in a water bath for 20 min, cooled in an ice bath, and centrifuged at 3000×*g* for 10 min. The absorbance of the supernatant and blank containing all reagents except the test drug was measured at 535 nm and expressed as TBARS- nmol/mg protein.

### 2.10. Histological Preparation

Following brain extraction, the tissue was stored in 4% paraformaldehyde solution, washed, and cut into 3 mm thick coronal sections using a sharp blade. Subsequently, these sections were fixed in paraffin blocks and sliced into 4 *μ*m thin coronal sections using a microtome [53]. These sections were processed using the following staining techniques.

### 2.11. Hematoxylin and Eosin Staining (H&E Staining)

Our previous lab protocols were used for H&E staining [54]. Briefly, tissue-coated slides were deparaffinized using absolute xylene followed by a graded alcohol solution. Next, the slides were stained by immersion in hematoxylin solution until the stain was retained in the nucleus. After treatment with 1% HCl and 1% ammonia water, the slides were treated with eosin solution for a few minutes and then air-dried. After dehydration with graded ethanol and xylene, as well as coverslipping, five images per slide were captured under an Olympus light microscope (Olympus, Japan) and analyzed using the ImageJ software. Histopathological changes in cellular morphology, shape, number, and edema were determined using light microscopy.

### 2.12. Immunohistochemical Analysis

Immunostaining was performed as previously discussed [55]. First, the tissue was rehydrated using xylene, graded alcohol series, and distilled water, followed by washing three times with PBS for 5 min. Proteinase K was used as the antigen recovery step. After washing, the tissue was treated with 3% H^2^O^2^ solution for 5 min to prevent endogenous peroxidase activity. Next, blocking serum was applied at room temperature for a minimum of 1 h to ensure blocking of areas outside the antigenic areas. The slides were then treated with anti-rabbit *Nrf2* antibody, anti-mouse VEGF antibody, anti-mouse *HO-1* antibody, anti-mouse p-NF-*κ*B antibody, and anti-mouse TNF-*α* antibody (dilution 1 : 100, Santa Cruz Biotechnology, Dallas, TX, USA) overnight at 4°C. The next day, the slides were initially treated with a secondary antibody for 2 h after washing with PBS. Next, the slides were treated with an ABC staining kit and left for 1 h. Finally, the slides were stained with DAB solution for 5 min, washed with water, dipped in xylene and 100% ethanol, and covered using mounting media. The slides were air-dried for a minimum of one day with images being obtained using an Olympus microscope and evaluated using ImageJ software. The slides were observed at 10x and 40x magnification; additionally, five random overlapping sections were chosen to calculate the number of stained neurons in the cortex and hippocampal CA1, CA2, and DG granule cells. The means were plotted against the groups.

### 2.13. ELISA (Enzyme-Linked Immunosorbent Assay)

COX-2, p-NF-*κ*B, and TNF-*α* expression were quantified using rat ELISA kits following the manufacturer's instructions. Briefly, an appropriate quantity of brain tissue (50 mg) was homogenized using a Heidolph crusher at 15,000 rpm in 2500 *μ*L PBS containing PMSF as the protease inhibitor [56]. Next, the tissue homogenate was centrifuged at 4000×g for 10 min, and the supernatant was collected. Total protein concentration in the supernatant of each group was calculated using the BCA method (Elabscience); moreover, an equivalent protein quantity was used to quantify the protein concentration of COX-2, p-NF-*κ*B, and TNF-*α* using an ELISA microplate reader (BioTek EL×808). Finally, the protein concentration (pg/mL) was normalized to the total protein content (pg/mg total protein).

### 2.14. Real-Time Polymerase Chain Reaction (RT-PCR)

TRIzol was used to extract the total RNA amount in freshly isolated brain tissues as previously discussed [57]. RNA quality and quantity were assessed using a NanoDrop plate (Skanit RE 4.1, Thermo Scientific). To convert RNA to cDNA, we used a viva cDNA synthesis kit (Vivantis cDSK01-050). Polymerase chain reactions were performed on a Galaxy XP Thermal Cycler (BIOER, PRC) and 2X Amplifyme Universal qPCR mix (Blirt, Germany), following the manufacturer's specifications. The sequences of forwarding and reverse primers were as follows: Rat_*Nrf2*_Forward: CACATCCAGACAGACACCAGT and Rat_*Nrf2*_Reverse: CTACAAATGGGAATGTCTCTGC; Rat_*HO-1*-Forward: CGTGCAGAGAATTCTGAGTTC and Rat_*HO-1*-Reverse: AGACGCTTTACGTAGTGCTG; Rat_GAPDH-Forward: CGTGCAGAGAATTCTGAGTTC and Rat_GAPDH-Reverse: TTCAGGTGAGCCCCAGCCTT. The relative gene expression of *Nrf2* was determined using the 2^−ΔΔCT^ method for real-time quantitative PCR.

### 2.15. Statistical Analysis

Statistical analysis was performed using the GraphPad prism-8 software. Neurobehavior and oxidative data were analyzed using one-way analysis of variance (ANOVA) followed by a post hoc Bonferroni multiple comparison test. The other data were interpreted using two-way ANOVA followed by post hoc Bonferroni multiple comparison tests. ImageJ software was used to analyze morphological data. Statistical significance was set at *p* < 0.05. Symbol ∗ or ^#^ represents *p* < 0.05; ^∗∗^ or ^##^ represents *p* < 0.01; and ^∗∗∗^ or ^###^ represents *p* < 0.001. Finally, ^∗^ and ^#^ represent significant differences relative to saline and disease, respectively. All data are expressed as the mean ± standard error of the mean (SEM).

## 3. Results

### 3.1. Acute Oral Toxicity Testing of Carveol

**Figure 3:**
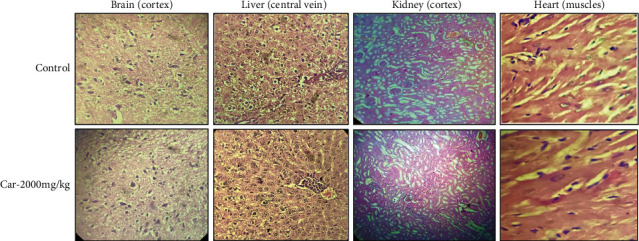
Data regarding acute oral toxicity showing histopathology of the control and carveol-treated groups at a limited dose (2000 mg/kg).

To assess the safety profile of carveol, OECD guidelines 425 were followed. Fur and skin, fecal consistency, urine color, respiration, and sleep patterns were found to be normal after administration of 2000 mg/kg of carveol. All animals in both groups survived, with none showing signs of convulsions or distress. Both groups showed normal weight progression during the 14-day protocol. There were no alterations in antioxidant profile, liver function tests, kidney function tests, and hematological indices (Suplementary Figures S1and S2). Histopathological screening of vital organs, including the brain, liver, kidney, and heart, revealed no signs of vacuolation, dystrophy, and/or atrophy (Figure 3). A detailed toxicity profile of carveol indicated that it was safe up to a dose of 2000 mg/kg.

### 3.2. Anticonvulsant Effect of Carveol on PTZ-Induced Seizure-Like Behavior

**Figure 4:**
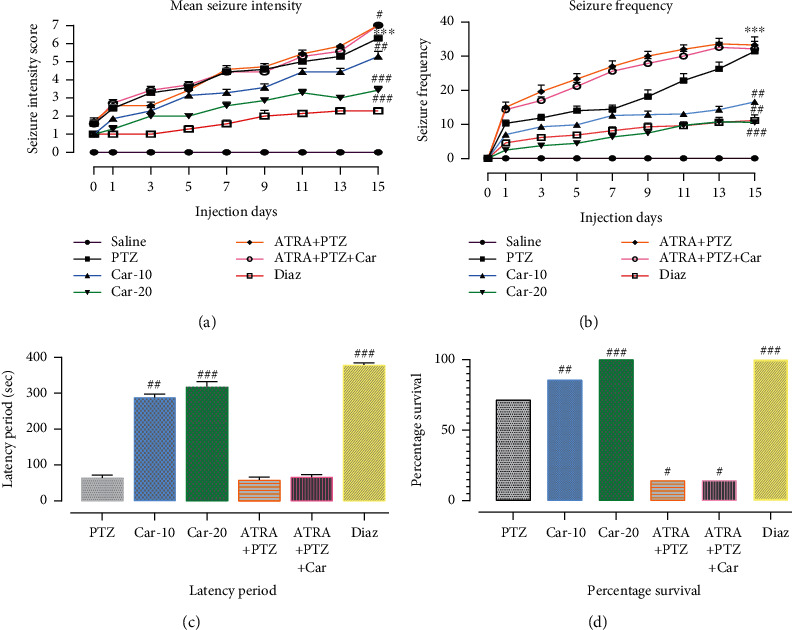
Effect of carveol on PTZ-induced seizure-like behavior. (a) The mean seizure intensity score was recorded after each PTZ injection. Each criterion was scored from 1 to 7. Carveol significantly reduced the mean seizure score as compared with PTZ-kindled animals. (b) Effect of carveol on seizure frequency. Seizure frequency is calculated as the number of tonic-clonic seizures within 30 min after PTZ injection. Car-20 displayed a significantly lower seizure frequency compared with kindled animals. (c) The latency period was measured as the duration between PTZ administration and the appearance of the first clonic seizure. Carveol displayed a delayed latency period compared with PTZ. (d) Compared with the saline group, the PTZ-kindled group showed a lower percentage of survival; moreover, Car-20 displayed an improved survival of the treated animals. All data were expressed as mean ± SEM (*n* = 10/group). # and ^∗^ denotes a significant difference compared with the PTZ-kindled and saline groups, respectively.

PTZ-treated animals presented significant generalized tonic-clonic convulsions also called epileptogenesis, and it corresponds to stage 6 and/or 7 of Racine's scale, as indicated by the significant mean seizure intensity score relative to the saline group (Figure 4(a), ^∗∗∗^*p* < 0.001). Similarly, PTZ significantly increased the seizure frequency on the 15^th^ day of administration (Figure 4(b), ^∗∗∗^*p* < 0.001) with a corresponding very short latency time, which depicts rapid seizure initiation on successive PTZ administration (Figure 4(c)). Overall, animals subjected to PTZ kindling exhibited a percentage survival of 71.4% (Figure 4(d)). Carveol treatment (10 mg/kg) reversed the PTZ-induced behavioral deficits, as indicated by the significantly low mean seizure intensity score (Figure 4(a), ^##^*p* < 0.01). Moreover, carveol significantly reduced seizure frequency (Figure 4(b), ^##^*p* < 0.01) and was associated with an extended latency time (Figure 4(c), ^##^*p* < 0.01). Additionally, the percentage survival improved to 85% compared with PTZ (Figure 4(d), ^##^*p* < 0.01). Similarly, carveol at a dose of 20 mg/kg showed similar protection given that none of the animals exhibited a seizure score of 4–5 (Figure 4(a), ^###^*p* < 0.001) during the whole kindling period; moreover, there was a significantly reduced seizure frequency (Figure 4(b), ^##^*p* < 0.01) and an extended latency period (Figure 4(c), ^###^*p* < 0.001). Diazepam showed similar protection to Car-20 (^###^*p* < 0.001). Moreover, cotreatment with PTZ and ATRA further aggravated seizure-like behavioral deficits, which could not be mitigated by carveol treatment, indicating the termination of carveol activity by ATRA administration.

### 3.3. Carveol Attenuated Cognitive Impairment and Memory Dysfunction in Epileptic Rats

**Figure 5:**
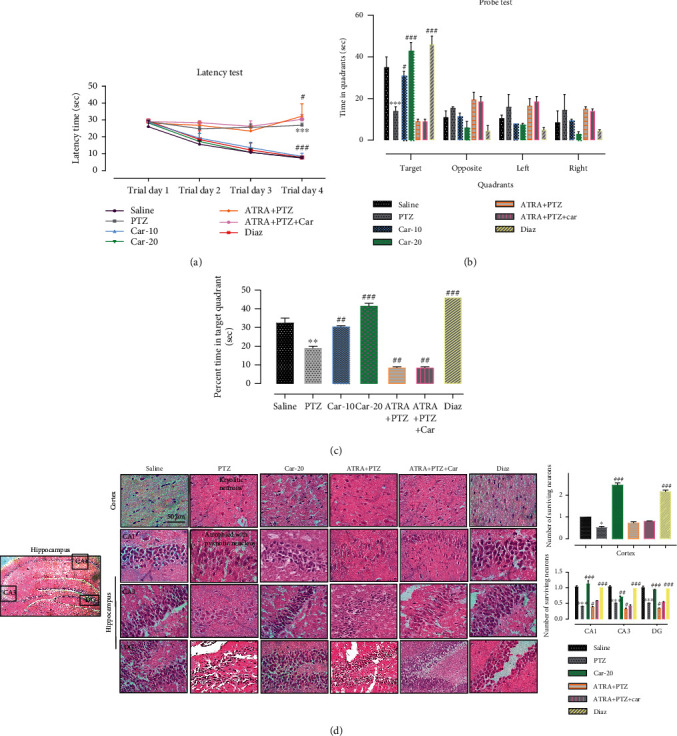
Effect of carveol on PTZ-induced memory impairment and neuronal survival. (a) The latency time of rats on the hidden platform. (b) Time spent by PTZ-treated rats in each quadrant in the probe test on the 5^th^ day. (c) The percentage time spent by animals in the target quadrant, with *n* = 10/group. (d) Representative photomicrographs of the H&E-stained cortex and hippocampal tissue revealing the presence of kryolitic and atrophied nuclei in PTZ-kindled animals while the Car-20 group showed only a few cells with degenerative signs (40x, scale bar 50 *μ*m). All data were expressed as mean ± SEM (*n* = 5/group). ^∗∗∗^*p* < 0.001 denotes a significant difference compared with the saline group. ^###^*p* < 0.001 denotes a significant difference compared with the PTZ-kindled group.

MWM test was used to assess the effect of carveol on memory and cognition in PTZ-induced epileptic rats. In the hidden-platform swimming test, PTZ-treated rats exhibited a higher latency time compared with saline-treated rats, which indicated severe memory deficits (Figure 5(a), ^∗∗∗^*p* < 0.001). Carveol treatment with 10 and 20 mg/kg doses significantly improved memory deficits and improved the latency time to reach the hidden platform (Figure 5(a), ^###^*p* < 0.001). To assess reference memory, a probe trial was conducted 24 h after the last acquisition period.  Figure 5(b) shows the time spent by each group of rats in specific quadrants. Increased time spent in quadrants other than the target quadrant is indicative of impaired spatial learning, as observed in the PTZ-kindled group (Figures 5(b) and 5(c), ^∗∗∗^*p* < 0.001, ^∗∗^*p* < 0.01). Upon treatment with 10 mg/kg carveol, the animals displayed significantly improved spatial memory and learning (Figures 5(b) and 5(c), ^#^*p* < 0.05, ^##^*p* < 0.01). Similarly, 20 mg/kg carveol significantly improved spatial memory and learning compared with the PTZ-treated animals (Figures 5(b) and 5(c), ^###^*p* < 0.001). Additionally, carveol treatment did not improve the ATRA-treated group, which indicated cessation of carveol activity by ATRA administration.

To further validate our hypothesis, we examined morphological changes in the cortical and hippocampal regions using H&E staining. The saline group showed round, well-demarcated intact cells without nuclear condensation or distortion with a basophilic cytoplasm (Figure 5(d)). The PTZ-treated group showed significant histopathological alterations, including altered neuronal shape and size, as well as other atypical features, including swollen, flattened, atrophied, and kryolitic neurons with pyknotic nuclei (Figure 5(d)). Examination of cortical and hippocampal areas confirmed that carveol (20 mg/kg) significantly ameliorated these morphological damages, as indicated by an increase in the number of intact neurons and cell count (Figure 5(d), cortex: ^###^*p* < 0.001, CA1 and DG: ^###^*p* < 0.001, CA3: ^##^*p* < 0.01). Additionally, carveol pretreatment in the ATRA-treated group did not improve PTZ-induced histopathological damage.

### 3.4. Carveol Augments the Antioxidant Capacity of the Brain through *Nrf2* and *Nrf2-*Dependant Downstream Antioxidant *HO-1*

**Figure 6:**
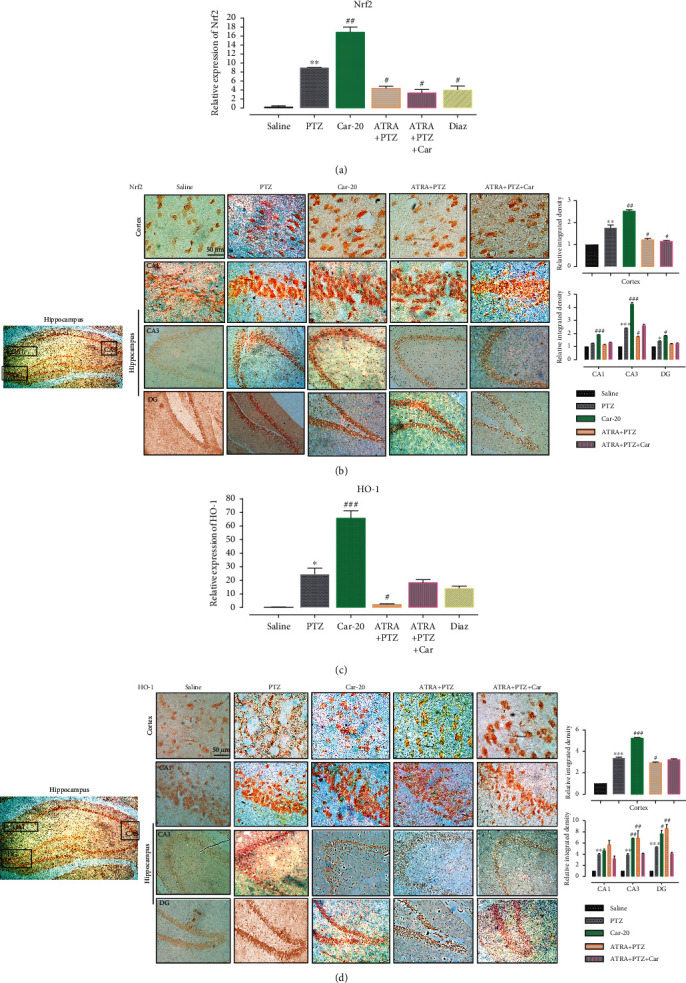
Carveol augments the antioxidant capacity of the brain via the *Nrf2* signaling pathway. (a) *Nrf2* gene expression as quantified by RT-PCR with (*n* = 5/group). (b) Immunohistochemistry results for *Nrf2* in the cortical and hippocampal tissues. Histograms exhibit higher *Nrf2* nuclear localization in treated brain tissues. Scale bar 50 *μ*m, magnification 40x (*n* = 5/group. (c) *HO-1* expression as quantified by RT-PCR with (*n* = 5/group). (d) Immunohistochemistry results for *HO-1* in the cortical and hippocampal tissues. Histograms exhibit higher *HO-1* nuclear localization in treated brain tissues. Scale bar 50 *μ*m, magnification 40x (*n* = 5/group). ^∗∗∗^*p* < 0.001 indicates significant difference relative to saline, while ^###^*p* < 0.001 shows significant difference compared with the PTZ group. All data are presented as means ± SEM.

*Nrf2* combines with free radicals and executes vital antioxidative functions. To further investigate the antioxidant potential of carveol, we analyzed the expression of the *Nrf2* gene and the downstream *HO-1*. RT-PCR analysis indicated upregulated *Nrf2* expression in the PTZ-treated group given that PTZ kindling exerted enough oxidative stress in the brain to activate the body's innate antioxidant *Nrf2* (Figure 6(a), ^∗∗^*p* < 0.01). To further validate this, immunohistochemistry revealed a notable upregulation (Figure 6(b)). Consistent with the upregulated *Nrf2* expression, there was a significant increase in the expression of the downstream antioxidant *HO-1* (Figure 6(c), ^∗^*p* < 0.05), also validated by immunohistochemistry (Figure 6(d)). Compared with PTZ, Carveol significantly upregulated hippocampal and cortical expression of *Nrf2* and *HO-1* (Figures 6(a)–6(d)). However, ATRA-treated groups exhibited blockade of carveol-mediated upregulation of the innate antioxidants *Nrf2* and *HO-1*, which suggested that the *Nrf2/HO-1* signaling pathway is involved in the antioxidant potential of carveol.

### 3.5. Carveol Ameliorates Inflammatory Mediators via the *Nrf2* Signaling Pathway

**Figure 7:**
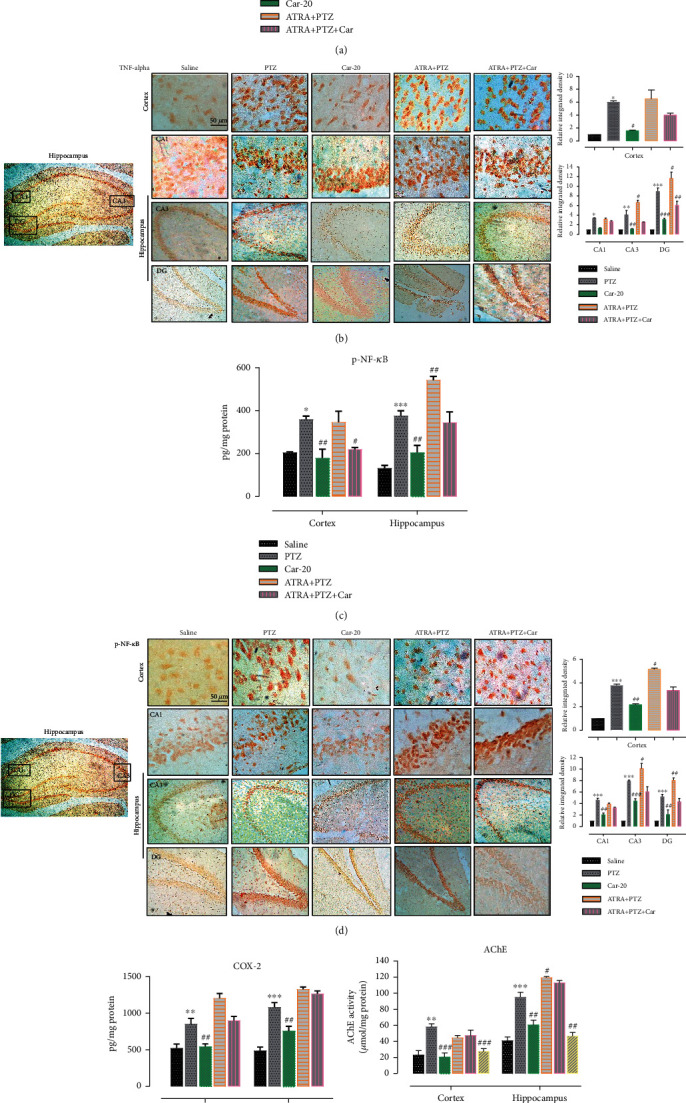
Effect of carveol on outcomes of PTZ-induced inflammatory mediators. (a) TNF-alpha protein expression as quantified by ELISA. (b) Immunohistochemistry results for TNF-alpha in the cortical and hippocampal tissues. TNF-*α* exhibited cytoplasmic localization in treated brain tissues. (c) p-NF-*_Κ_*B protein expression as quantified by ELISA. (d) Immunohistochemistry results for p-NF-*_Κ_*B in the cortical and hippocampal tissues. p-NF-*_Κ_*B exhibited nucleus localization in the treated tissue. (e) COX-2 protein expression as quantified by ELISA. (f) Acetylcholinesterase levels in both the cortex and hippocampal tissue. The data were expressed as the mean ± SEM, *n* = 5/group in each experiment. ^∗∗∗^*p* < 0.001 shows differences compared with saline while ^####^*p* < 0.001 shows significant differences compared with PTZ. Scale bar 50 *μ*m, magnification 40x.

Numerous studies have shown that PTZ-kindling is associated with an intensified inflammatory cascade. Therefore, we proceeded to determine whether carveol treatment could affect neuroinflammation. TNF-*α*, an inflammatory cytokine, was highly expressed in the kindled model as evaluated both by ELISA (Figure 7(a), cortex: ^∗^*p* < 0.05, hippocampus: ^∗^*p* < 0.05) and by immunohistochemistry (Figure 7(b), cortex, CA1: ^∗^*p* < 0.05; CA3: ^∗∗^*p* < 0.01; DG: ^∗∗∗^*p* < 0.001). As part of downstream targets, we evaluated p-NF-*κ*B and COX-2 expression. The PTZ-treated group showed elevated p-NF-*κ*B expression as shown by ELISA (Figure 7(c), cortex: ^∗^*p* < 0.05, hippocampus: ^∗∗∗^*p* < 0.001) and by immunohistochemistry (Figure 7(d), ^∗∗∗^*p* < 0.001). A similar expression pattern was also observed for COX2 by ELISA (Figure 7(e), cortex: ^∗∗^*p* < 0.01, hippocampus: ^∗∗∗^*p* < 0.001).

Additionally, AChE levels were measured given the involvement of the cholinergic system in neuroinflammation. Compared with the saline group, the PTZ-treated group showed a significant upregulation of AChE levels (Figure 7(f), cortex: ^∗∗^*p* < 0.01, hippocampus: ^∗∗∗^*p* < 0.001). Carveol pretreatment remarkably attenuated expression of TNF-*α* (Figures 7(a) and 7(b)), p-NF-*κ*B (Figures 7(c) and 7(d)), COX-2 (Figure 7(e)), and AChE (Figure 7(f)) in both the cortex and hippocampus. When cotreatment with ATRA and PTZ exaggerated the neuroinflammatory markers, carveol treatment could not reverse the deleterious effects of PTZ in the ATRA-treated groups.

### 3.6. Carveol Improves BBB Disruption through Growth Factors

**Figure 8:**
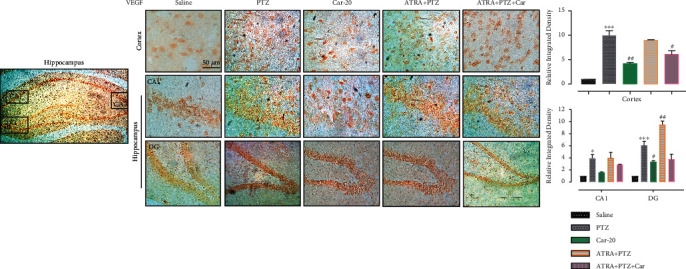
Carveol improves BBB-disruption through VEGF. Immunohistochemistry results for VEGF in the cortical and hippocampal tissues exhibited cytoplasmic localization in both brain tissues. Data are expressed as the mean ± SEM, *n* = 5. ^∗∗∗^*p* < 0.001 shows difference compared with saline while ^####^*p* < 0.001 shows significant difference compared with PTZ. Scale bar 50 *μ*m, magnification 40x.

Previous studies have demonstrated the induction of growth factors such as vascular endothelial growth factor (VEGF) in seizures due to BBB disruption. Consistently, our study demonstrated abrupt VEGF induction following epileptiform activity in PTZ-treated animals compared with the saline group (Figure 8, cortex: ^∗∗∗^*p* < 0.001, CA1: ^∗^*p* < 0.05, DG: ^∗∗∗^*p* < 0.001). Carveol attenuated VEGF hyperexpression in all brain regions, which successfully restored brain permeability (Figure 8, cortex: ^##^*p* < 0.01, DG: ^#^*p* < 0.05). On the other hand, ATRA treatment significantly induced angiogenic factor VEGF, which resulted in BBB dysfunction and diminished the restorative potential of carveol in the cortex of ATRA-treated groups (Figure 8, cortex: ^#^*p* < 0.05).

### 3.7. Effect of Carveol on PTZ-Induced Lipid Oxidation and Oxidative Stress Markers

**Figure 9:**
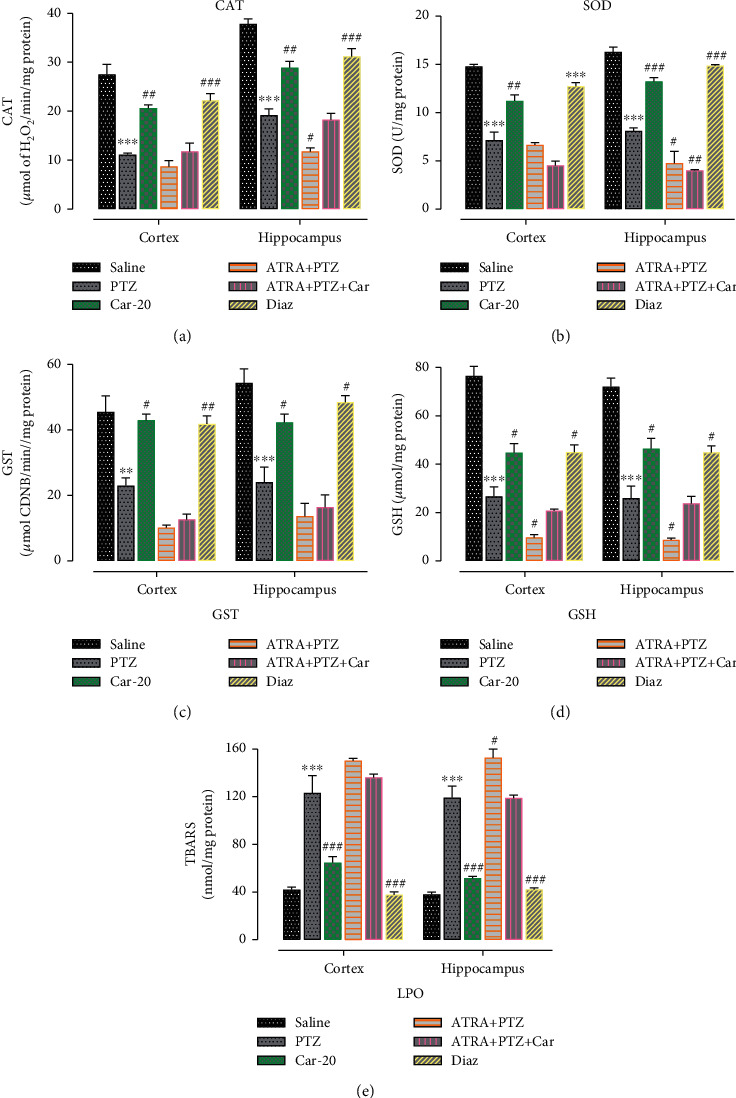
Effect of carveol on oxidative enzymes (Catalase, SOD, GST, GSH, and LPO) in the cortex and hippocampus. (a) CAT level. (b) SOD level. (c) GST level. (d) GSH level. (e) LPO level. ^∗∗∗^*p* < 0.001 denotes a significant difference compared with the saline group. ^###^*p* < 0.001 denotes significant differences compared with the PTZ group. *n* = 7/group. Data are expressed as mean ± SEM.

To assess the neuroprotective potential of carveol against PTZ-induced oxidative stress markers, we measured the cortical and hippocampal levels of various enzymatic and nonenzymatic antioxidants, including SOD, CAT, GST, GSH, and TBARS (Figure 9). Carveol treatment significantly restored the level of these antioxidants in the cortex and hippocampus to varying degrees. Compared with the saline group, the PTZ-treated group showed significantly lower levels of CAT, SOD, GST, and GSH (Figures 9(a)–9(d), ^∗∗∗^*p* < 0.001). Contrastingly, compared with the saline group, the PTZ-treated group showed a marked elevation in LPO levels (Figure 9(e), ^∗∗∗^*p* < 0.001). Carveol treatment significantly increased cortical and hippocampal levels of CAT, SOD, GST, and GSH (Figure 9(a), ^##^*p* < 0.01; Figure 9(b), cortex: ^##^*p* < 0.01, hippocampus: ^###^*p* < 0.001; Figure 9(c), ^#^*p* < 0.05; Figure 9(d), ^#^*p* < 0.05). Conversely, carveol-treated animals demonstrated noticeably reduced TBARS levels in both the cortex and hippocampus compared with the PTZ group (Figure 9(e), ^###^*p* < 0.001). Moreover, carveol treatment in ATRA-treated groups demonstrated minimal or no antioxidant effects (Figure 9), which is consistent with our previous findings (Figure 6).

## 4. Discussion

Plant-derived natural compounds are consistently employed against different pathological disorders owing to better treatment options and minimum side effects. This is due to their inherent properties of targeting multiple steps in the pathological cascade. For this purpose, extensive research must be conducted on phytochemicals before human clinical trials as neuroprotectants [58]. Previous studies have shown that carveol is a monoterpene possesses robust antioxidant, anti-inflammatory, and protective properties in various degenerative models [33, 59]. However, there have been no direct reports regarding the antiepileptic potential of carveol. This study investigated the neuroprotective potential of carveol in a PTZ-induced chronic epilepsy model by ameliorating cognitive deficits, oxidative stress, and neuroinflammation. Our findings showed that carveol had significant potential in reverting seizures by augmenting the endogenous *Nrf2* antioxidant pathway.

In this study, we used subconvulsive PTZ doses, which is a well-studied chemical inducer of epileptic seizures that exerts proconvulsant activity through GABAergic inhibition and therefore causes an imbalance in inhibitory and excitatory neurotransmission that induces seizures [19, 60, 61]. Carveol treatment attenuated these seizures by decreasing the seizure intensity and frequency, as well as delaying seizure onset. Hippocampal dysfunction and neuronal hyperexcitability in epileptic seizures are directly associated with various memory and cognitive impairments [62]. Our findings were consistent with previous findings as a decline in cognition and memory impairment in PTZ-treated animals was demonstrated by a significant increase in escape latency and the probe test [63]. However, carveol significantly improved memory deficits, as demonstrated by a shorter latency time and greater time spent in the target quadrant.

Several studies have reported the involvement of ROS in the pathophysiology of neurodegenerative diseases including epilepsy [64, 65]. Likewise, oxidative stress can exacerbate epilepsy as the brain has limited antioxidants combating capacity [66]. Moreover, the degree of oxidative damage is proportional to epileptic episodes [67]. This notion is further supported by the fact that several clinically used antiepileptic drugs (AEDs) alleviated ROS in seizure [68], while many other AEDs exacerbated oxidative damage [69, 70]. Therefore the use of adjunct antioxidants with AEDs can be useful in the management of epilepsy as demonstrated previously [71]. Our results were consistent with those of previous studies where PTZ-kindled animals experienced oxidative stress and revealed diminished levels of SOD, CAT, GST, and GSH [72, 73]. Carveol augmented these antioxidants and reduced LPO levels, which may partly account for its neuroprotective ability. The substantial oxidative stress caused by seizures activates the endogenous antioxidant response pathway, *Nrf2*, and therefore increases the expression of cytoprotective enzymes and ROS scavengers [74, 75]. The *Nrf2* pathway forms an important defense against oxidative insults in both glial cells and neurons [76–78]. Using *Nrf2*-knockout mice, Wang et al. concluded that the *Nrf2*-ARE pathway is directly involved in protecting the brain from seizure-mediated neuronal damage [79, 80]. In another study, Mazzuferi et al. used gene expression datasets and observed exaggerated *Nrf2* mRNA levels in the hippocampus of mice that initiated spontaneous recurrent seizures [81]. Additionally, Li et al. confirmed the involvement of the *Nrf2*-ARE pathway through nuclear *Nrf2* translocation and direct ARE activation [82]. Another study suggested a strong correlation between ARE activation and *HO-1* expression [83]. Our findings are consistent with these previous findings and we demonstrated upregulated *Nrf2* expression along with the downstream inducible *HO-1* gene and protein in PTZ-treated animals. Moreover, carveol treatment led to further augmentation of these genes and protein levels. However, *Nrf2* inhibition through ATRA abolished these effects, indicating the strong involvement of the *Nrf2* pathway in the cytoprotective nature of carveol.

*Nrf2* pathway activation inhibits proinflammatory cytokine release and downregulates the p-NF-*κ*B pathway [26, 27]. Numerous studies have suggested the role of inflammatory mediators such as COX-2 and p-NF-*κ*B activation along with other inflammatory cytokines including interleukin-6 (IL-6), interleukin-1 (IL-1b), and TNF-*α* that form the basis of neuronal injury in several neurodegenerative models including epilepsy [11, 84, 85]. Furthermore, studies have suggested increased COX-2 induction in the mouse brain after electrical kindling of hippocampal pyramidal cells [10]. Carveol significantly ameliorated the upregulated inflammatory mediators in PTZ-treated animals, which is consistent with recent findings regarding the modulatory effect of carveol on proinflammatory cytokines [33, 59]. ATRA treatment further exacerbated the expression of inflammatory markers and abolished the anti-inflammatory potential of carveol, which supports our hypothesis that carveol exerts its anti-inflammatory potential by modulating the *Nrf2* pathway.

Moreover, the cholinergic anti-inflammatory pathway is significantly involved in the modulation of immune response and inflammation in the brain [86]. This notion is further supported by the fact that several acetylcholinesterase (AChE) inhibitors promoted anti-inflammatory activities [82, 87]. Studies have suggested that the local immune response and inflammation are associated with the upregulation of hippocampal AChE levels, resulting in cholinergic imbalance and epileptogenesis [86]. Studies have shown that AChE may be an important therapeutic target for adjunct treatment in epilepsy as numerous AChE-inhibitors were tested in experimental settings for this purpose as a memory enhancer [88–90]. Moreover, our findings suggested increased brain AChE level, which was significantly inhibited by carveol treatment, and indicated a modulating effect of carveol on cholinergic transmission.

Studies have shown the association between vascular malformations and epilepsy, which suggested that subsequent BBB dysfunction could induce neuronal hyperactivity. Additionally, BBB leakage is associated with excessive angiogenesis induced by VEGF, and this expression is increased in patients with temporal lobe epilepsy [91]. Similarly, altered membrane fluidity and enhanced permeability caused by BBB disruption in epileptic tissue cause angiogenesis and dysfunctional vascular permeability [92]. Additionally, our experimental findings suggested upregulated VEGF expression in epileptic animals, which was ameliorated by carveol treatment, indicating an improvement in angiogenesis caused by PTZ.

## 5. Conclusions

**Figure 10:**
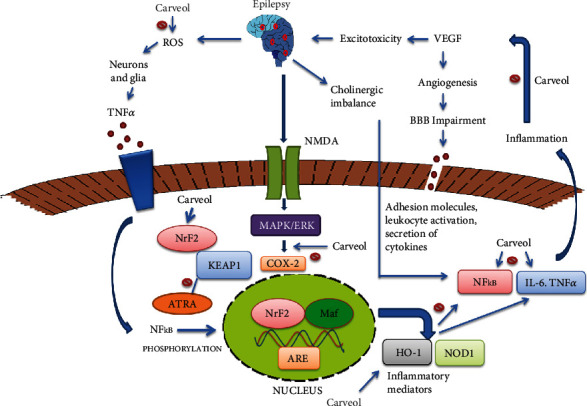
Diagrammatic illustration elaborating the underlying antioxidant and neuroprotective potential of carveol in a PTZ-induced epilepsy model.

In conclusion, our findings demonstrated that carveol could be a potent antioxidant and anti-inflammatory drug candidate that can exert neuroprotection in a PTZ-induced animal epilepsy model. We also demonstrated certain safety aspects of carveol, and it exhibited a relative safety profile as no impairment was observed in the kidneys, heart, liver, and brain further assisted by biochemical analysis. Furthermore, we demonstrated the involvement of the *Nrf2*-pathway in the neuroprotective activity of carveol (Figure 10). Additionally, we observed the potential of carveol to negatively modulate inflammatory mediators, angiogenic factors, and cholinergic imbalance; however, still, further experimentation is required to unveil its exact mechanism in epilepsy.

## Figures and Tables

**Table 1:** Modified Racine's scale.

Stages	Seizure intensity
0	No response
1	Hyperactivity, restlessness, and vibrissae twitching
2	Head nodding, head clonus, and myoclonic jerks
3	Unilateral or bilateral limb clonus
4	Forelimb clonic seizures
5	Generalized clonic seizures with falling
6	Hind limb extensor
7	Death

## Data Availability

The research data used to support the findings of this study are included within the article.

## Supplementary Materials

Supplementary Materials Supplementary 1 Supplementary Figure S1: effect of carveol at 2000 mg/kg on the antioxidant enzymes of the brain. Supplementary 2. Supplementary Figure S2: effect of carveol at 2000 mg/kg on (a) liver enzymes, (b) renal function, (c) weights of organs, and (d) hematological indices.

## References

[1] Moshé S. L., Perucca E., Ryvlin P., Tomson T. (2015). Epilepsy: new advances. *The Lancet*.

[2] Baradaran Rahimi V., Askari V. R., Hosseini M., Yousefsani B. S., Sadeghnia H. R. (2019). Anticonvulsant activity of viola tricolor against seizures induced by pentylenetetrazol and maximal electroshock in mice. *Iranian journal of medical sciences*.

[3] Waldbaum S., Patel M. (2010). Mitochondrial dysfunction and oxidative stress: a contributing link to acquired epilepsy?. *Biomembranes*.

[4] Gnatek Y., Zimmerman G., Goll Y., Najami N., Soreq H., Friedman A. (2012). Acetylcholinesterase loosens the brain's cholinergic anti-inflammatory response and promotes epileptogenesis. *Frontiers in Molecular Neuroscience*.

[5] Walsh S., Donnan J., Fortin Y. (2017). A systematic review of the risks factors associated with the onset and natural progression of epilepsy. *NeuroToxicology*.

[6] Löscher W., Klitgaard H., Twyman R. E., Schmidt D. (2013). New avenues for anti-epileptic drug discovery and development. *Nature Reviews Drug Discovery*.

[7] Gaitatzis A., Sander J. W. (2013). The long-term safety of antiepileptic drugs. *CNS Drugs*.

[8] Kumar S., Sarangi S. C., Tripathi M., Gupta Y. K. (2020). Evaluation of adverse drug reaction profile of antiepileptic drugs in persons with epilepsy: a cross-sectional study. *Epilepsy & Behavior*.

[9] Ho Y.-H., Lin Y. T., Wu C. W. J., Chao Y. M., Chang A. Y. W., Chan J. Y. H. (2015). Peripheral inflammation increases seizure susceptibility via the induction of neuroinflammation and oxidative stress in the hippocampus. *Journal of Biomedical Science*.

[10] Vezzani A., Granata T. (2005). Brain inflammation in epilepsy: experimental and clinical evidence. *Epilepsia*.

[11] Vezzani A., French J., Bartfai T., Baram T. Z. (2011). The role of inflammation in epilepsy. *Nature Reviews Neurology*.

[12] Cardenas-Rodriguez N., Huerta-Gertrudis B., Rivera-Espinosa L. (2013). Role of oxidative stress in refractory epilepsy: evidence in patients and experimental models. *International Journal of Molecular Sciences*.

[13] Bhat A. H., Dar K. B., Anees S. (2015). Oxidative stress, mitochondrial dysfunction and neurodegenerative diseases; a mechanistic insight. *Biomedicine & Pharmacotherapy*.

[14] Alvarez J. I., Teale J. M. (2006). Breakdown of the blood brain barrier and blood-cerebrospinal fluid barrier is associated with differential leukocyte migration in distinct compartments of the CNS during the course of murine NCC. *Journal of Neuroimmunology*.

[15] Aronica E., Crino P. B. (2011). Inflammation in epilepsy: clinical observations. *Epilepsia*.

[16] Vezzani A., French J., Bartfai T., Baram T. Z. (2011). The role of inflammation in epilepsy. *Nature Reviews. Neurology*.

[17] Righes Marafiga J., Vendramin Pasquetti M., Calcagnotto M. E. (2020). GABAergic interneurons in epilepsy: more than a simple change in inhibition. *Epilepsy & Behavior*.

[18] Ullah I., Badshah H., Naseer M. I., Lee H. Y., Kim M. O. (2015). Thymoquinone and vitamin C attenuates pentylenetetrazole-induced seizures via activation of GABAB1 receptor in adult rats cortex and hippocampus. *Neuromolecular Medicine*.

[19] Huang R.-Q., Bell-Horner C. L., Dibas M. I., Covey D. F., Drewe J. A., Dillon G. H. (2001). Pentylenetetrazole-induced inhibition of recombinant *γ*-aminobutyric acid type A (GABAA) receptors: mechanism and site of action. *Journal of Pharmacology and Experimental Therapeutics*.

[20] Dhir A. (2012). Pentylenetetrazol (PTZ) kindling model of epilepsy. *Current Protocols in Neuroscience*.

[21] Shin E.-J., Jeong J. H., Chung Y. H. (2011). Role of oxidative stress in epileptic seizures. *Neurochemistry International*.

[22] Bashkatova V., Narkevich V., Vitskova G., Vanin A. (2003). The influence of anticonvulsant and antioxidant drugs on nitric oxide level and lipid peroxidation in the rat brain during penthylenetetrazole-induced epileptiform model seizures. *Progress in Neuro-Psychopharmacology and Biological Psychiatry*.

[23] Ambrogini P., Torquato P., Bartolini D. (2019). Excitotoxicity, neuroinflammation and oxidant stress as molecular bases of epileptogenesis and epilepsy-derived neurodegeneration: the role of vitamin E. *Biochimica et Biophysica Acta (BBA) - Molecular Basis of Disease*.

[24] Kasai S., Shimizu S., Tatara Y., Mimura J., Itoh K. (2020). Regulation of Nrf2 by mitochondrial reactive oxygen species in physiology and pathology. *Biomolecules*.

[25] Zhu H., Itoh K., Yamamoto M., Zweier J. L., Li Y. (2005). Role of Nrf2 signaling in regulation of antioxidants and phase 2 enzymes in cardiac fibroblasts: protection against reactive oxygen and nitrogen species-induced cell injury. *FEBS Letters*.

[26] Kensler T. W., Wakabayashi N., Biswal S. (2007). Cell survival responses to environmental stresses via the Keap1-Nrf2-ARE pathway. *Annual Review of Pharmacology and Toxicology*.

[27] Bellezza I., Tucci A., Galli F. (2012). Inhibition of NF-*κ*B nuclear translocation via HO-1 activation underlies *α*-tocopheryl succinate toxicity. *The Journal of Nutritional Biochemistry*.

[28] Buendia I., Michalska P., Navarro E., Gameiro I., Egea J., León R. (2016). Nrf2-ARE pathway: an emerging target against oxidative stress and neuroinflammation in neurodegenerative diseases. *Pharmacology & Therapeutics*.

[29] Fagiani F., Catanzaro M., Buoso E. (2020). Targeting cytokine release through the differential modulation of Nrf2 and NF-*κ*B pathways by electrophilic/non-electrophilic compounds. *Frontiers in Pharmacology*.

[30] Munguía-Martínez M. F., Nava-Ruíz C., Ruíz-Díaz A., Díaz-Ruíz A., Yescas-Gómez P., Méndez-Armenta M. (2019). Immunohistochemical study of antioxidant enzymes regulated by Nrf2 in the models of epileptic seizures (KA and PTZ). *Oxidative Medicine and Cellular Longevity*.

[31] al-Sawaf O., Clarner T., Fragoulis A. (2015). Nrf2 in health and disease: current and future clinical implications. *Clinical science*.

[32] Lim J. L., Wilhelmus M. M. M., de Vries H. E., Drukarch B., Hoozemans J. J. M., van Horssen J. (2014). Antioxidative defense mechanisms controlled by Nrf2: state-of-the-art and clinical perspectives in neurodegenerative diseases. *Archives of Toxicology*.

[33] Malik I., Shah F. A., Ali T. (2020). Potent natural antioxidant carveol attenuates MCAO-stress induced oxidative, neurodegeneration by regulating the Nrf-2 pathway. *Frontiers in Neuroscience*.

[34] Mohsin Alvi A., Tariq al Kury L., Umar Ijaz M. (2020). Post-treatment of synthetic polyphenolic 1,3,4 oxadiazole compound A3, attenuated ischemic stroke-induced neuroinflammation and neurodegeneration. *Biomolecules*.

[35] Naeem K., Tariq al Kury L., Nasar F. (2021). Natural dietary supplement, carvacrol, alleviates LPS-induced oxidative stress, neurodegeneration, and depressive-like behaviors via the Nrf2/HO-1 pathway. *Journal of Inflammation Research*.

[36] Shah F. A., Kury L. A., Li T. (2019). Polydatin attenuates neuronal loss via reducing neuroinflammation and oxidative stress in rat MCAO models. *Frontiers in Pharmacology*.

[37] Ali J., Khan A. U., Shah F. A. (2019). Mucoprotective effects of Saikosaponin-A in 5-fluorouracil-induced intestinal mucositis in mice model. *Life Sciences*.

[38] Decarvalho C., Dafonseca M. (2006). Carvone: why and how should one bother to produce this terpene. *Food Chemistry*.

[39] Crowell P. L., Kennan W. S., Haag J. D., Ahmad S., Vedejs E., Gould M. N. (1992). Chemoprevention of mammary carcinogenesis by hydroxylated derivatives of d-limonene. *Carcinogenesis*.

[40] Sachan A. K., Das D. R., Kumar M. (2016). Carum carvi-an important medicinal plant. *Journal of Chemical and Pharmaceutical Research*.

[41] Rahman Z. U., al Kury L. T., Alattar A. (2021). Carveol a naturally-derived potent and emerging nrf2 activator protects against acetaminophen-induced hepatotoxicity. *Frontiers in Pharmacology*.

[42] Ahmed M. S., Khan A. U., Kury L. T. A., Shah F. A. (2020). Computational and pharmacological evaluation of carveol for antidiabetic potential. *Frontiers in Pharmacology*.

[43] Awotunde O. S., Adewoye S. O., Dhanabal P. S., Hawumba J. (2019). Subacute toxicity study of aqueous root extract of *Terminalia schimperiana* in male Wistar rats. *Toxicology Reports*.

[44] Saleem U., Amin S., Ahmad B., Azeem H., Anwar F., Mary S. (2017). Acute oral toxicity evaluation of aqueous ethanolic extract of *Saccharum munja* Roxb. roots in albino mice as per OECD 425 TG. *Toxicology Reports*.

[45] Guzzo E. F. M., Lima K. R., Vargas C. R., Coitinho A. S. (2018). Effect of dexamethasone on seizures and inflammatory profile induced by Kindling Seizure Model. *Journal of Neuroimmunology*.

[46] Dhir A., Naidu P. S., Kulkarni S. K. J. S. (2007). Neuroprotective effect of nimesulide, a preferential COX-2 inhibitor, against pentylenetetrazol (PTZ)-induced chemical kindling and associated biochemical parameters in mice. *Seizure*.

[47] Vieira V., Glassmann D., Marafon P., Pereira P., Gomez R., Coitinho A. S. (2016). Effect of diclofenac sodium on seizures and inflammatory profile induced by kindling seizure model. *Epilepsy Research*.

[48] Ellman G. L., Courtney K. D., Andres V., Featherstone R. M. (1961). A new and rapid colorimetric determination of acetylcholinesterase activity. *Biochemical Pharmacology*.

[49] al Kury L., Zeb A., Abidin Z. U. (2019). Neuroprotective effects of melatonin and celecoxib against ethanol-induced neurodegeneration: a computational and pharmacological approach. *Drug Design, Development and Therapy*.

[50] Imran M., al Kury L. T., Nadeem H. (2020). Benzimidazole containing acetamide derivatives attenuate neuroinflammation and oxidative stress in ethanol-induced neurodegeneration. *Biomolecules*.

[51] Zulfiqar Z., Shah F. A., Shafique S. (2020). Repurposing FDA approved drugs as JNK3 inhibitor for prevention of neuroinflammation induced by MCAO in rats. *Journal of Inflammation Research*.

[52] al Kury L. T., Dayyan F., Ali Shah F. (2020). *Ginkgo biloba* extract protects against methotrexate-induced hepatotoxicity: a computational and pharmacological approach. *Molecules*.

[53] Ullah U., Badshah H., Malik Z. (2020). Hepatoprotective effects of melatonin and celecoxib against ethanol-induced hepatotoxicity in rats. *Immunopharmacology and Immunotoxicology*.

[54] Iqbal S., Shah F. A., Naeem K. (2020). Succinamide derivatives ameliorate neuroinflammation and oxidative stress in scopolamine-induced neurodegeneration. *Biomolecules*.

[55] Irshad N., Khan A. U., Shah F. A. (2021). Antihyperlipidemic effect of selected pyrimidine derivatives mediated through multiple pathways. *Fundamental & Clinical Pharmacology*.

[56] Firdous A., Sarwar S., Shah F. A. (2021). Contribution of attenuation of TNF-*α* and NF-*κ*B in the anti-epileptic, anti-apoptotic and neuroprotective potential of *Rosa webbiana* fruit and its chitosan encapsulation. *Molecules*.

[57] Imran M., Shah F. A., Nadeem H. (2021). Synthesis and biological evaluation of benzimidazole derivatives as potential neuroprotective agents in an ethanol-induced rodent model. *ACS Chemical Neuroscience*.

[58] Kumar G. P., Khanum F. (2012). Neuroprotective potential of phytochemicals. *Pharmacognosy Reviews*.

[59] Rahman Z. U., al Kury L. T., Alattar A. (2021). Carveol a naturally-derived potent and emerging Nrf2 activator protects against acetaminophen-induced hepatotoxicity. *Frontiers in Pharmacology*.

[60] Löscher W. J. S. (2011). Critical review of current animal models of seizures and epilepsy used in the discovery and development of new antiepileptic drugs. *Seizure*.

[61] Naseer M., Ullah N., Ullah I. (2011). Vitamin C protects against ethanol and PTZ-induced apoptotic neurodegeneration in prenatal rat hippocampal neurons. *Synapse*.

[62] Cardoso A., Lukoyanova E. A., Madeira M. D., Lukoyanov N. V. (2011). Seizure-induced structural and functional changes in the rat hippocampal formation: comparison between brief seizures and status epilepticus. *Behavioural Brain Research*.

[63] Ahmadian S. R., Ghasemi-Kasman M., Pouramir M., Sadeghi F. (2019). Arbutin attenuates cognitive impairment and inflammatory response in pentylenetetrazol-induced kindling model of epilepsy. *Neuropharmacology*.

[64] Liu Z., Zhou T., Ziegler A. C., Dimitrion P., Zuo L. (2017). Oxidative stress in neurodegenerative diseases: from molecular mechanisms to clinical applications. *Oxidative Medicine and Cellular Longevity*.

[65] Cardenas-Rodriguez N., Huerta-Gertrudis B., Rivera-Espinosa L. (2013). Role of oxidative stress in refractory epilepsy: evidence in patients and experimental models. *International Journal of Molecular Sciences*.

[66] Freitas R. M. (2009). Investigation of oxidative stress involvement in hippocampus in epilepsy model induced by pilocarpine. *Neuroscience Letters*.

[67] Varoglu A. O., Yildirim A., Aygul R., Gundogdu O. L., Sahin Y. N. (2010). Effects of valproate, carbamazepine, and levetiracetam on the antioxidant and oxidant systems in epileptic patients and their clinical importance. *Clinical Neuropharmacology*.

[68] Ueda Y., Doi T., Takaki M., Nagatomo K., Nakajima A., Willmore L. J. (2009). Levetiracetam enhances endogenous antioxidant in the hippocampus of rats: _In vivo_ evaluation by brain microdialysis combined with ESR spectroscopy. *Brain Research*.

[69] Karikas G. A., Schulpis K. H., Bartzeliotou A. (2009). Early effects of sodium valproate monotherapy on serum paraoxonase/arylesterase activities. *Scandinavian Journal of Clinical and Laboratory Investigation*.

[70] Verrotti A., Scardapane A., Franzoni E., Manco R., Chiarelli F. (2008). Increased oxidative stress in epileptic children treated with valproic acid. *Epilepsy Research*.

[71] Uma Devi P., Kolappa Pillai K., Vohora D. (2006). Modulation of pentylenetetrazole-induced seizures and oxidative stress parameters by sodium valproate in the absence and presence of N-acetylcysteine. *Fundamental and Clinical Pharmacology*.

[72] Méndez-Armenta M., Nava-Ruíz C., Juárez-Rebollar D., Rodríguez-Martínez E., Yescas Gómez P. (2014). Oxidative stress associated with neuronal apoptosis in experimental models of epilepsy. *Oxidative Medicine and Cellular Longevity*.

[73] Goel R., Saxena P. (2019). Pycnogenol protects against pentylenetetrazole-induced oxidative stress and seizures in mice. *Current Clinical Pharmacology*.

[74] Motohashi H. (2004). Nrf2–Keap1 defines a physiologically important stress response mechanism.

[75] Buendia I. (2016). Nrf2–ARE pathway: an emerging target against oxidative stress and neuroinflammation in neurodegenerative diseases.

[76] Sandberg M. (2014). NRF2-regulation in brain health and disease: implication of cerebral inflammation.

[77] Dinkova-Kostova A. T., Abramov A. Y. (2015). The emerging role of Nrf2 in mitochondrial function. *Free Radical Biology and Medicine*.

[78] Sivandzade F., Prasad S., Bhalerao A., Cucullo L. (2019). NRF2 and NF-қB interplay in cerebrovascular and neurodegenerative disorders: molecular mechanisms and possible therapeutic approaches. *Redox biology*.

[79] Wang W., Wu Y., Zhang G. (2014). Activation of Nrf2-ARE signal pathway protects the brain from damage induced by epileptic seizure. *Brain research*.

[80] Wang W., Wang W. P., Zhang G. L. (2013). Activation of Nrf2-ARE signal pathway in hippocampus of amygdala kindling rats. *Neuroscience Letters*.

[81] Mazzuferi M., Kumar G., van Eyll J., Danis B., Foerch P., Kaminski R. M. (2013). Nrf2 defense pathway: experimental evidence for its protective role in epilepsy. *Annals of neurology*.

[82] Pavlov V. A., Parrish W. R., Rosas-Ballina M. (2009). Brain acetylcholinesterase activity controls systemic cytokine levels through the cholinergic anti-inflammatory pathway. *Brain, behavior, and immunity*.

[83] Kietzmann T., Samoylenko A., Immenschuh S. (2003). Transcriptional Regulation of Heme Oxygenase-1 Gene Expression by MAP Kinases of the JNK and p38 Pathways in Primary Cultures of Rat Hepatocytes. *Journal of Biological Chemistry*.

[84] Verrotti A., Latini G., Scardapane A., Manco R., Vecchio A. (2007). The role of inflammation in epilepsy. *Current Pediatric Reviews*.

[85] Ali A., Shah F. A., Zeb A. (2020). NF-*κ*B inhibitors attenuate MCAO induced neurodegeneration and oxidative stress—a reprofiling approach. *Frontiers in molecular neuroscience*.

[86] Gnatek Y., Zimmerman G., Goll Y., Najami N., Soreq H., Friedman A. (2012). Acetylcholinesterase loosens the brain's cholinergic anti-inflammatory response and promotes epileptogenesis. *Frontiers in molecular neuroscience*.

[87] Nizri E., Irony-Tur-Sinai M., Faranesh N. (2008). Suppression of neuroinflammation and immunomodulation by the acetylcholinesterase inhibitor rivastigmine. *Journal of neuroimmunology*.

[88] Tonduli L., Testylier G., Masqueliez C., Lallement G., Monmaur P. (2001). Effects of huperzine used as pre-treatment against soman-induced seizures. *Neurotoxicology*.

[89] Mishra A., Goel R. K. (2014). Adjuvant anticholinesterase therapy for the management of epilepsy-induced memory deficit: a critical pre-clinical study. *Basic & clinical pharmacology & toxicology*.

[90] Getova D., Dimitrova D. S. (2000). Effects of the anticholinesterase drug tacrine on the development of PTZ kindling and on learning and memory processes in mice. *Folia medica*.

[91] Ogaki A., Ikegaya Y., Koyama R. (2020). Vascular abnormalities and the role of vascular endothelial growth factor in the epileptic brain. *Frontiers in pharmacology*.

[92] Morin-Brureau M., Rigau V., Lerner-Natoli M. J. E. (2012). Why and how to target angiogenesis in focal epilepsies. *Epilepsia*.

