# Supplementary material for: Carveol Attenuates Seizure Severity and Neuroinflammation in Pentylenetetrazole-Kindled Epileptic Rats by Regulating the Nrf2 Signaling Pathway
Source: Oxid Med Cell Longev. 2021 Aug 11;2021:9966663. doi: 10.1155/2021/9966663 (PMC8376446; doi:10.1155/2021/9966663)

**Carveol attenuates seizure severity and neuroinflammation in pentylenetetrazole-kindled epileptic rats by regulating the *Nrf2* signaling pathway**

# Supplementary Material


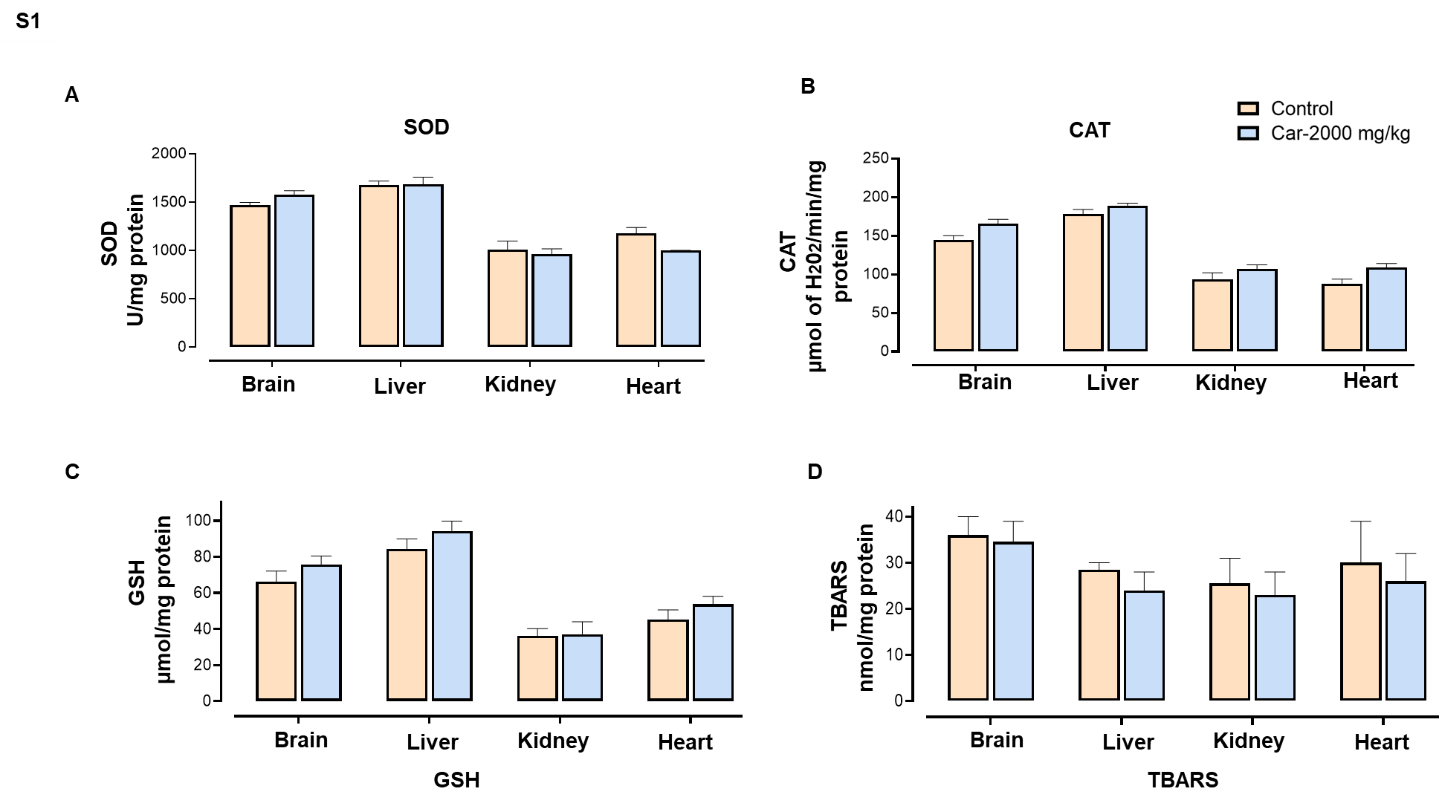


S1: Effect of carveol at 2000 mg/kg on the antioxidant enzymes of the brain


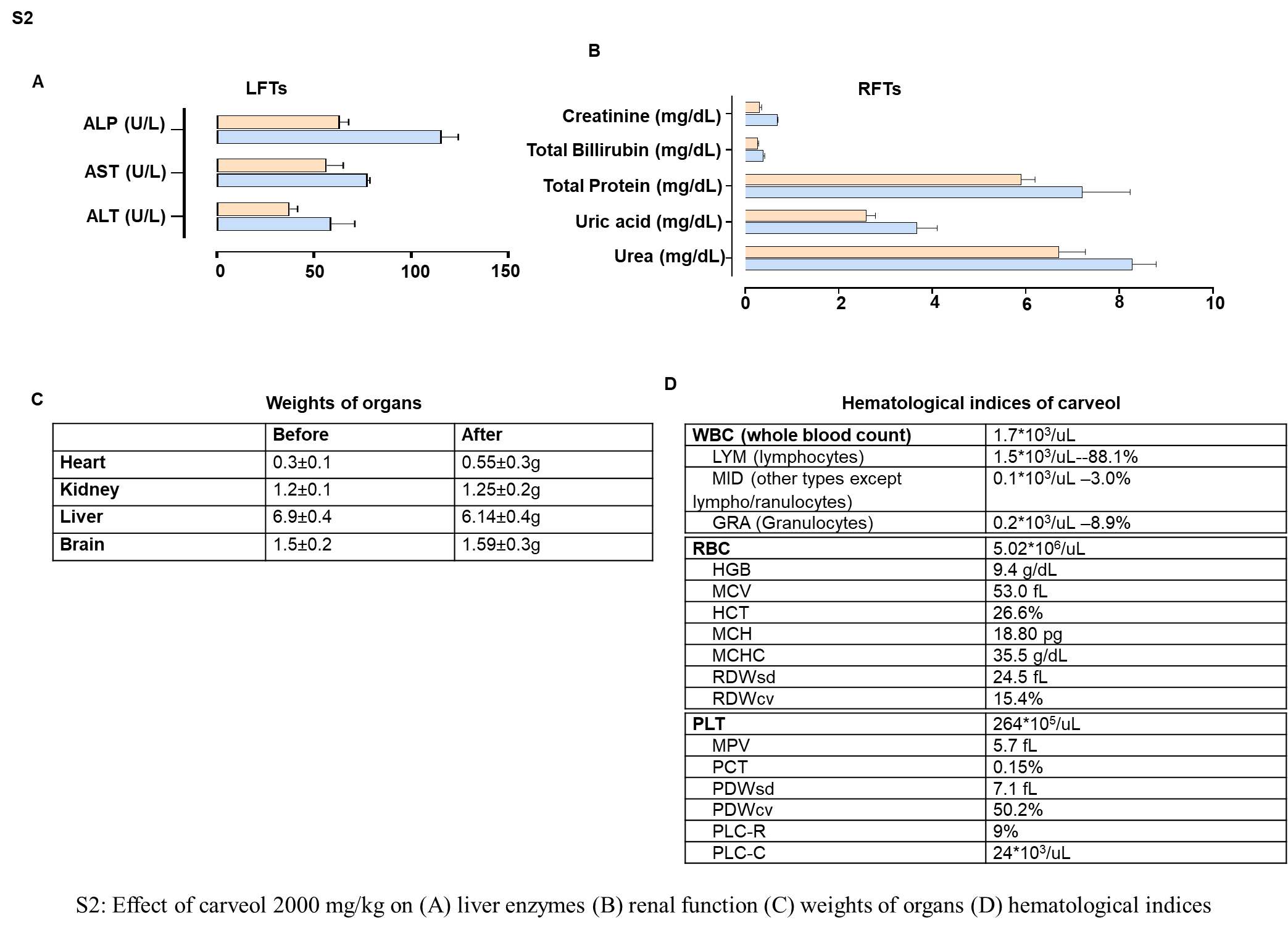

Supplement: Supplementary Materials — Supplementary 1 Supplementary Figure S1: effect of carveol at 2000 mg/kg on the antioxidant enzymes of the brain. Supplementary 2. Supplementary Figure S2: effect of carveol at 2000 mg/kg on (a) liver enzymes, (b) renal function, (c) weights of organs, and (d) hematological indices. [file 9966663.f1.docx]
